# Using the Foresight 50+ National Probability Panel of Older Adults for Rapid Recruitment for Qualitative Research

**DOI:** 10.1093/geroni/igaf122.123

**Published:** 2025-12-31

**Authors:** Ellen Bloss, Nell Compernolle, Martha Cowley, Alex Chew

**Affiliations:** NORC at the University of Chicago, Chicago, Illinois, United States; NORC at the University of Chicago, Chicago, Illinois, United States; National Opinion Research Center at The University of Chicago, Chicago, Illinois, United States; National Opinion Research Center at The University of Chicago, Chicago, Illinois, United States

## Abstract

**Background and Objectives:**

The older adult population is rapidly growing. However, they remain severely underrepresented in research, in large part due to recruitment challenges. As a result, aging research is often constrained by large recruitment budgets or unrepresentative convenience samples. Here, we tested the feasibility of a novel, high quality recruitment tool for rapid qualitative research for an NIH-funded pilot study on loneliness among older adults.

**Methods:**

We leveraged the Foresight 50 + (“F50+”), a sub-panel of AmeriSpeak that is offered by AARP and NORC and fields monthly Omnibus surveys to a nationally representative sample of U.S. adults ages 50 and older. As part of the F50+ Omnibus survey (fielded to N∼1000 panelists), we included the 3-item UCLA loneliness scale and an item assessing their interest in participating in a 60-minute focus group discussion.

**Results:**

Coupled with the F50+ Profile variables, we used the Omnibus results to identify U.S. adults who are ages 65 + (N = 602), reported some loneliness (N = 292), were willing to participate in a group discussion (N = 92), and varied across key socio-demographic characteristics (e.g., gender and race/ethnicity). The study team scheduled and successfully conducted 4 virtual focus group discussions with our target population, the compositions of which varied by gender and race/ethnicity, within 4 weeks at a reasonable cost. In general, panelists were agreeable, engaging, and communicative, with minimal attrition or invalid contact information.

**Discussion:**

The Foresight 50+ panel offers a cost-effective option for researchers and policymakers seeking high quality participants for rapid research among sub-populations of older adults.

